# Identification of novel immunogens in *Pasteurella multocida*

**DOI:** 10.1186/1475-2859-6-3

**Published:** 2007-01-18

**Authors:** Keith Al-Hasani, John Boyce, Victoria P McCarl, Stephen Bottomley, Ian Wilkie, Ben Adler

**Affiliations:** 1 Australian Research Council Centre of Excellence in Structural and Functional Microbial Genomics, Monash University, Victoria 3800, Australia; 2 School of Veterinary Science, University of Queensland, Queensland 4072, Australia

## Abstract

*P. multocida *is a Gram-negative pathogen responsible for causing diseases in animals of economic significance to livestock industries throughout the world. Current vaccines include bacterins, which provide only limited protection against homologous serotypes. Therefore there is a need for more effective vaccines to control diseases caused by *P. multocida*. As a step towards developing vaccines against fowl cholera, a genomics based approach was applied for the identification of novel immunogens.

**Results:**

Bioinformatics analysis of the *P. multocida *genome predicted 129 proteins as secreted, located in the outer membrane, or lipoproteins. 105 of the genes encoding these proteins were cloned and recombinant protein expressed in *Escherichia coli*. Polyclonal serum from *P. multocida*-infected chickens reacted with a subset of these proteins.

**Conclusion:**

These data show the range of bacterial immunogens recognized by the chicken immune system, including 6 novel immunoreactive proteins.

## Background

*Pasteurella multocida *is a Gram-negative bacterial pathogen which is the causative agent of a range of diseases in animals, including fowl cholera in avian species, hemorrhagic septicemia in ungulates, shipping fever and pneumonia in cattle, atrophic rhinitis in swine, and snuffles in rabbits [1-3]. The bacterium also causes infection in humans, primarily through dog and cat bites. Fowl cholera, which is generally caused by serotypes A:1, A:3 or A:4 [4], is a severe systemic disease which occurs in domestic poultry and wild birds and results in significant economic losses to poultry industries worldwide. Current vaccines against fowl cholera include bacterins [5], which provide only limited protection against homologous serotypes and live attenuated strains, which have been observed to revert to virulence [6]. Therefore, there is a need for more effective vaccines to control diseases caused by *P. multocida*.

The surface of Gram-negative bacteria is critical for interaction of the bacterium with the host cell environment as it mediates nutrient uptake, secretion of toxins and other products and is involved in avoidance of the host immune system [7]. Furthermore, it is the bacterial surface molecules that are the targets for host immunity. Indeed, bacterial surface proteins have been shown to be important for conferring protective immunity in a range of infection models [8,9]. Recently, the *P. multocida *PlpB protein was identified as a cross-protective antigen [10,11] and this protein is located in the *P. multocida *outer membrane [12]. Outer membrane proteins also promote adherence to host cell surfaces and are therefore likely to be involved in *P. multocida *virulence [13].

In an effort to identify novel immunogens to which chickens respond during natural infection, and as a first step towards developing protective recombinant vaccines against fowl cholera, a detailed antigen profiling analysis was undertaken. The effectiveness of this approach, which has been termed 'reverse vaccinology' or 'reverse immunology', has been reported by several authors with *Plasmodium falciparum *[14], *Streptococcus pneumoniae *[15], *Treponema pallidum *[16], *Neisseria meningitidis *[17], and *Chlamydia pneumoniae *[18].

## Results and discussion

**Figure 1:**
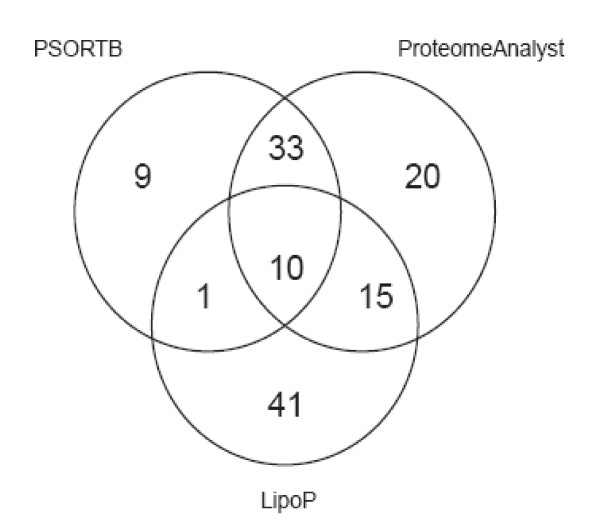
Venn diagram showing the different bioinformatics prediction of the outer membrane and secreted proteins from the *P. multocida *Pm70 genome using the algorithms, PSORTB (19) (outer membrane or extracellular), ProteomeAnalyst (20) (outer membrane or secreted) and LipoP (21) (SpII cleavage site predicted).

We utilised a range of bioinformatics analyses of the annotated *P. multocida *Pm70 genome sequence and previously published experimental data [12] to select genes likely to have vaccine potential. The central premise of this work was that protective antigens are likely to be surface exposed or secreted by the bacteria and therefore accessible to the host immune response. We used PSORTB [19] and ProteomeAnalyst [20] to predict all outer membrane and secreted proteins and LipoP [21] to predict all lipoproteins. We included all lipoproteins as a large number of lipoproteins was observed in the proteomics analysis of the *P. multocida *outer membrane [12] and we believe that precise sub-cellular prediction of lipoprotein location (inner or outer membrane) is presently unreliable. These bioinformatics analyses identified a combined list of 129 proteins (Figure 1, see Additional file 1). The prediction programs PSORTB and ProteomeAnalyst can be used to predict protein localisation in Gram-negative organisms, while LipoP specifically predicts Gram-negative lipoproteins. PSORTB and ProteomeAnalyst use different methods for prediction of localisations. PSORTB uses primary sequence analysis algorithms and gives a high precision, but low sensitivity, prediction. Proteome analyst on the other hand utilises analysis of text annotations for the closest homologue of any given query sequence and is medium-high precision and sensitivity; it thus makes a higher number of predictions. These analyses were complemented by the previously published proteomic analysis of the *P. multocida *outer membrane [12] which identified three proteins (PM0612, PM1357 and PM1746) not predicted by bioinformatics.

The large number of candidate genes necessitated the adoption of a high-throughput cloning strategy, which utilised by the Gateway™trogen Inc., Carlsbad, CA) cloning and expression system to clone PCR-amplified *P. multocida *ORFs. An attempt was made to clone all 129 ORFs; however, only 105 were successfully cloned and expressed in *E. coli*. Each of the recombinant expression clones was assessed for levels of protein expression and solubility. Because of the large inventory of proteins that were required to be expressed and purified in a time and cost effective way, protein expression was analysed using a novel inclusion body assay developed on a TECAN liquid handling robot.

**Figure 2:**
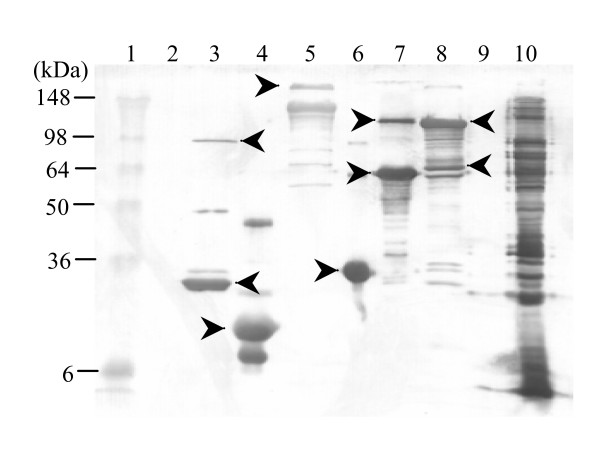
Western immunoblot demonstrating recognition of six novel *P. multocida *antigens by pooled sera from experimentally infected chickens. Sizes of markers in kilodaltons are indicated on the left. Lanes: 1, protein molecular standards (kDa) (SeeBlue Plus2 Prestained Sandard, Novagen); 2 and 9 are blank; 3, PM0442 (pLIC-Nus); 4, PM0979 (pDEST-17); 5, PM0659 (pDEST-17); 6, PM1993 (pDEST-17); 7, PM1614 (pLIC-Nus); 8, PM1979 (pLIC-Nus); 10, *P. multocida *X73 whole cell lysate. All the soluble fusion proteins cloned with a NusA tag were cleaved and detected after incubation with Tev protease at room temperature for 2 h. The top arrows in the corresponding lanes show the expected size of the fusion proteins and the bottom arrows mark the recombinant proteins without the NusA tag. Lanes with single arrows correspond to the recombinant proteins expressed in the absence of a NusA tag.

To evaluate the range of *P. multocida *proteins recognised by the chicken antibody response, serum samples from chickens that had been repeatedly infected with *P. multocida *and then treated with antibiotics were tested for reactivity against 105 recombinant proteins by Western blot. Pooled serum samples from *P. multocida *infected chickens (8 from X-73, 3 from VP161 – a highly virulent A:1 strain [22]) and four serum samples from control chickens were reacted in immunoblot assays with comparable amounts (3 μg/lane) of recombinant proteins. The immune chicken sera generated during *P. multocida *strain X-73 or VP161 infection recognised a total of 12 (Table 1) recombinant proteins, ten of which were not recognised by naive sera. To our knowledge, six of these antigens have not been previously identified as capable of eliciting immune responses from chickens, demonstrating the utility of this approach for the identification of novel immunoreactive bacterial antigens (Figure 2). Although immunological reactivity to unique antigens alone does not necessarily indicate a role in protective immunity against pasteurellosis, these novel antigens constitute prime candidates as potential vaccine targets for pasteurellosis, and warrant inclusion in studies to elucidate their potential role in pathogensis. The results have important implications in understanding avian immune responses to *P. multocida *and in elucidating putative elements of a protective immune response.

**Table 1:** *P. multocida *antigens recognised by immune chicken sera. +, reaction; -, no reaction.

**Antigen**	**Database similarity (accession no.)**	**X-73 sera**	**VP161 sera**	**Control sera**
PM0388	Outer membrane protein from *P. multocida *(AAK02472)	+	+	-
PM0442	Hypothetical protein	+	+	-
PM0554	Lipoprotein from *P. multocida *(AAK02638)	-	+	-
PM0659	Hypothetical protein	+	+	-
PM0786	OmpA from *P. multocida *(AAT57679)	+	+	+
PM0966	Omp16 from *P. multocida *(CAB75338)	+	+	+
PM0979	Hypothetical protein	+	+	-
PM1614	Outer membrane protein from *Haemophilus *(AAX87732)	+	+	-
PM1730	PlpB from *P. multocida *(AAK03814)	-	+	-
PM1979	SurA protein from *Mannheimia *(AAU38450)	+	+	-
PM1992	Oma87 from *P. multocida *(CAD20126)	+	+	-
PM1993	Outer membrane protein from *P. multocida *serotype D (CAA52400)	+	+	-

One drawback of this immunogen discovery assay is that it is limited to protein coding regions that can be expressed in *E. coli*. In addition, antibodies that recognise only conformational epitopes that are denatured in SDS-PAGE will not be detected. Furthermore, a lack of antibody recognition to recombinant proteins may be the result of a poor immune response to these antigens during infection or alternatively, a reflection of differential expression of antigens by the VP161 and X-73 strains within the host. Four of the six novel proteins (PM0442, PM0659, PM0979, PM1614) were identified as putative lipoproteins characterised by a cysteine residue after a signal peptide. PM1993 was predicted to contain a signal peptide processed by signal peptidase 1. These results do not preclude the existence of additional immunogens present in other serotypes.

## Conclusion

In summary, this work represents a systematic and comprehensive approach for the characterisation of the immune response associated with pasteurellosis by targeted computational screening of genomic sequences with predictive antigenic properties, followed by an automated high throughput expression and purification strategy using a robotic platform. It has resulted in the identification of 12 immunogenic determinants which are clearly expressed *in vivo *during infection, six of which were unique to this study, and thus constitute candidates for the development of a protective vaccine against fowl cholera. We are currently evaluating the protective effects of these *P. multocida *proteins against bacterial challenge in chickens.

## Methods

### Cloning of P.multocida genes

The Pm70 genome sequence was used when designing primers for X-73, which is virulent for chickens, while Pm70 does not cause disease in chickens. PCR primers were designed to amplify the gene region encoding the mature length protein (excluding the signal sequence) except where a signal sequence could not be predicted and then the primers were designed to encompass the entire gene. All 5' PCR primers included a 5'-CACC tail to facilitate directional topoisomerase cloning and where the primers had been designed to amplify only the mature length portion (without the signal sequence) the 5'-CACCATG tail was added so as to include a start codon. As the genes were to be expressed in-frame with either a C-terminal or an N-terminal tag the native stop codon was not included in the reverse primers. All PCR products were amplified from *P. multocida *strain X-73 (serotype A:1), which is highly virulent for chickens, and cloned into the Gateway entry vector pENTR/SD/D-TOPO^® ^(Invitrogen Inc. Carlsbad, CA). After the cloned genes were verified by sequencing and restriction digestion analysis, they were transferred by recombination (LR Clonase kit – Invitrogen Inc., Carlsbad, CA) from the entry clone to the Invitrogen destination vectors pBAD-DEST49™, pDEST-17™ and a Gateway adapted expression vector containing a NusA solubility tag, pLIC-Nus [23].

### Protein expression

Briefly, 1 ml of each culture (Overnight Express™, Novagen, Madison, WI) was chemically lysed with PopCulture (Novagen, Madison, WI), and Lysonase™ (Novagen, Madison, WI). The cell lysate was then added to a 96-well filter plate (AcroPrep™, EastHills, NY) and the solution was drawn through under vacuum. The inclusion bodies were retained while soluble proteins passed through the filter. The retained inclusion bodies were washed once with Triton X-100 to remove any remaining soluble proteins, followed by two washes with phosphate buffer. The washed proteins were then denatured by the addition of 200 μl of 8 M urea to each corresponding well, incubated for 2 hr at room temperature and collected under vacuum. Both soluble and insoluble fractions were then analysed by SDS-PAGE to assess the solubility of each protein.

### Immunoblotting

The membranes were incubated with sera (diluted 1:500) from either infected or uninfected birds as the primary antibody and then with a peroxidase-conjugated anti-chicken antibody (diluted 1:1000) as the secondary antibody (Chemicon International Inc., Temecula, CA). 4-chloro-1-napthol was used as the chromogenic detection reagent.

## Supplementary Material

Additional File 1 Bioinformatics analysis of 129 predicted *P. multocida *proteins. The data provided represent proteins that are predicted to be secreted, located in the outer membrane, or lipoproteins. Click here for file
