# Supplementary material for: Identification of novel immunogens in Pasteurella multocida
Source: Microb Cell Fact. 2007 Jan 18;6:3. doi: 10.1186/1475-2859-6-3 (PMC1781955; doi:10.1186/1475-2859-6-3)
Supplement: Additional File 1 — Bioinformatics analysis of 129 predicted P. multocida proteins. The data provided represent proteins that are predicted to be secreted, located in the outer membrane, or lipoproteins. [file 1475-2859-6-3-S1.doc]

Additional file 1. List of *in silico* predicted outer membrane and secreted proteins from the *P. multocida* Pm70 genome using the algorithms, PSORTB (8) (outer membrane or extracellular), ProteomeAnalyst (12) (outer membrane or secreted), and LipoP (11) (SpII cleavage site predicted).

| Gene Name | Pm Number | PSORTB prediction | Proteome Analyst prediction | LipoP prediction | Description |
| --- | --- | --- | --- | --- | --- |
| *PM0016* | PM0016 | Unknown | Inner membrane | SpII | hypothetical HI099 |
| *PM0022* | PM0022 | Unknown | Periplasm | SpII | hypothetical HI0178 |
| *dsbE_2* | PM0028 | Periplasmic | Periplasm | SpII | thiol:disulfide interchange protein |
| *pfhR* | PM0040 | Outer membrane | Outer membrane | SpI | Outer membrane heme receptor |
| *PM0055* | PM0055 | Unknown | Secreted | CYT | hypothetical HI0282 |
| *lspB_1* | PM0056 | Outer membrane | Outer membrane | SpI | hemolysin accessory protein |
| *pfhB1* | PM0057 | Outer membrane | Secreted | SpII | putative virulence factor, filamentous hemaglutinin |
| *lspB_2* | PM0058 | Outer membrane | Outer membrane | SpI | hemolysin accessory protein |
| *pfhB2* | PM0059 | Outer membrane | Secreted | SpII | putative virulence factor, filamentous hemaglutinin |
| *PM0067* | PM0067 | Unknown | Inner membrane | SpII | hypothetical HI0983 |
| *PM0072* | PM0072 | Unknown | Periplasm | SpII | hypothetical HI0960 |
| *est* | PM0076 | Outer membrane | Secreted | SpI | Outer membrane esterase |
| *oapA* | PM0098 | Extracellular | Secreted | CYT | opacity associated protein |
| *PM0243* | PM0243 | Unknown | Secreted | SpI | hypothetical HI0409 |
| *lolB* | PM0246 | Unknown | Outer membrane | SpII | Outer membrane lipoprotein |
| *PM0300* | PM0300 | Outer membrane | Outer membrane | SpI | TonB-dependent receptor, putative |
| *PM0305* | PM0305 | Outer membrane | No prediction | CYT | unknown |
| *ompW* | PM0331 | Outer membrane | Outer membrane | SpI | Outer membrane protein ompW precursor |
| *PM0336* | PM0336 | Outer membrane | Outer membrane | SpI | TonB-dependent receptor, putative |
| *PM0337* | PM0337 | Outer membrane | Outer membrane | SpI | Hemoglobin/haptoglobin binding protein |
| *PM0355* | PM0355 | Cytoplasmic | Secreted | CYT | esterase/lipase, putative |
| *PM0368* | PM0368 | Extracellular | Inner membrane | SpI | hypothetical HI1625 |
| *ompH_1* | PM0388 | Outer membrane | Outer membrane | SpI | Outer membrane protein |
| *ompH_2* | PM0389 | Outer membrane | Outer membrane | SpI | Outer membrane protein |
| *PM0442* | PM0442 | Inner membrane | Inner membrane | SpII | unknown |
| *PM0445* | PM0445 | Unknown | Secreted | CYT | pyrimidine dimer-specific glycoslyase |
| *PM0513* | PM0513 | Inner membrane | Periplasm | SpII | lytic transglycosylase |
| *PM0527* | PM0527 | Outer membrane | Outer membrane | SpII | Outer membrane channel protein, putative |
| *glgx* | PM0542 | Cytoplasmic | Secreted | CYT | glycogen operon protein |
| *PM0553* | PM0553 | Unknown | Periplasm | SpII | unknown |
| *lpp* | PM0554 | Inner membrane | Outer membrane | SpII | 15 kDa peptidoglycan-associated lipoprotein |
| *hemR* | PM0576 | Outer membrane | Outer membrane | SpII | heme-hemopexin utilization protein C |
| *plp4* | PM0586 | Outer membrane | Outer membrane | SpII | lipoprotein plp4 |
| *pepN* | PM0618 | Cytoplasmic | Secreted | CYT | aminopeptidase N |
| *PM0627* | PM0627 | Unknown | Periplasm | SpII | hypothetical HI1314, lipoprotein |
| *ponC* | PM0644 | Inner membrane | Inner membrane | SpII | bifunctional penicillin-binding protein 1C |
| *lppC* | PM0646 | Unknown | Inner membrane | SpII | lipoprotein LppC |
| *PM0649* | PM0649 | Periplasmic | Periplasm | SpII | hemolysin, putative |
| *PM0659* | PM0659 | Outer membrane | Outer membrane | SpII | hypothetical R. prowazekii |
| *PM0663* | PM0663 | Outer membrane | Periplasm | CYT | sialidase precursor, neuraminidase |
| *PM0674* | PM0674 | Unknown | Periplasm | SpII | unknown |
| *pdpB* | PM0676 | Inner membrane | Outer membrane | TMH | Phosphatidylglycero-phosphatase B |
| *PM0678* | PM0678 | Periplasmic | Periplasm | SpII | oligopeptide-binding protein homolog, Periplasmic, putative |
| *prlC* | PM0680 | Cytoplasmic | Secreted | CYT | oligopeptidase A |
| *PM0698* | PM0698 | Outer membrane | cytoplasm | CYT | methyltransferase, type III DNA modification enzyme |
| *PM0699* | PM0699 | Unknown | Secreted | CYT | type III restriction enzyme, restriction endonuclease |
| *PM0708* | PM0708 | Unknown | Outer membrane | SpII | putative Outer membrane protein, HI0389 |
| *PM0709* | PM0709 | Unknown | Secreted | CYT | O-sialoglycoprotein endopeptidase, putative |
| *hsf_1* | PM0714 | Unknown (This protein may have multiple localalization sites.) | Secreted | CYT | hsf homolog, locus encoding surface fibrils |
| *PM0741* | PM0741 | Outer membrane | Outer membrane | SpI | hemoglobin receptor precursor |
| *PM0745* | PM0745 | Unknown | Outer membrane | CYT | Outer membrane heme receptor, hypothetical |
| *PM0758* | PM0758 | Unknown | No prediction | SpII | unknown |
| *hexD* | PM0778 | Outer membrane | Outer membrane | SpII | HexD, capsule biosynthetic locus |
| *PM0786* | PM0786 | Outer membrane | Outer membrane | SpI | Outer membrane protein |
| *PM0803* | PM0803 | Outer membrane | Outer membrane | SpI | hypothetical HI1369 |
| *pqqL* | PM0804 | Unknown | Periplasm | SpII | zinc protease, zinc |
| *ompH_3* | PM0831 | Outer membrane | Outer membrane | CYT | Outer membrane protein |
| *tadD* | PM0846 | Unknown | No prediction | SpII | TadD, nonspecific tight adherence protein |
| *rcpA* | PM0852 | Outer membrane | Outer membrane | SpI | rough colony protein A |
| *PM0881* | PM0881 | Unknown | Secreted | SpII | nuclease, putative, HI1296 |
| *impA* | PM0892 | Unknown | Secreted | CYT | ImpA protein, putative |
| *PM0903* | PM0903 | Outer membrane | Secreted | SpI | N-acetylmuramoyl-L-alanine amidase |
| *PM0928* | PM0928 | Unknown | Secreted | SpII | membrane-bound lytic murein transglycosylase A |
| *PM0931* | PM0931 | Unknown | No prediction | SpII | unknown |
| *PM0966* | PM0966 | Outer membrane | Outer membrane | SpII | Outer membrane protein |
| *PM0979* | PM0979 | Unknown | Periplasm | SpII | unknown |
| *PM0982* | PM0982 | Unknown | No prediction | SpII | unknown |
| *PM0998* | PM0998 | Outer membrane | Outer membrane | SpI | unknown |
| *PM0999* | PM0999 | Unknown | Secreted | CYT | pre-B cell enhancing factor-related protein |
| *wza* | PM1016 | Outer membrane | Outer membrane | SpII | polysacchardie export protein wza |
| *opa* | PM1025 | Outer membrane | Outer membrane | SpI | opacity protein, putative |
| *PM1044* | PM1044 | Unknown | Periplasm | SpII | hypothetical, A. actinmyc. |
| *PM1050* | PM1050 | Unknown | Outer membrane | SpII | hypothetical HI0256 |
| *PM1060* | PM1060 | Unknown | Inner membrane | SpII | hypothetical HI0650 |
| *PM1064* | PM1064 | Unknown | Outer membrane | SpII | lipoprotein |
| *PM1069* | PM1069 | Outer membrane | Outer membrane | SpI | Outer membrane protein P1 precursor |
| *PM1073* | PM1073 | Unknown (This protein may have multiple localization sites.) | Inner membrane | SpII | hypothetical HI0966 |
| *PM1077* | PM1077 | Unknown | Outer membrane | SpII | lipoprotein |
| *PM1081* | PM1081 | Outer membrane | Outer membrane | SpI | iron-regulated outer membran protein, putative |
| *PM1113* | PM1113 | Unknown | Secreted | CYT | lipoprotein, putative, NlpI homolog |
| *PM1176* | PM1176 | Unknown | Periplasm | SpII | unknown |
| *PM1190* | PM1190 | Unknown | Inner membrane | SpII | hypothetical, N. meningitidis |
| *PM1215* | PM1215 | Unknown | Periplasm | SpII | lipoprotein, putative |
| *comE* | PM1225 | Outer membrane | Outer membrane | SpI | competence E protein |
| *gcp* | PM1238 | Extracellular | Secreted | CYT | O-sialoglycoprotein endopeptidase |
| *PM1282* | PM1282 | Outer membrane | No prediction | SpI | Outer membrane hemin receptor, putative |
| *mltC* | PM1321 | Periplasmic | Periplasm | SpII | membrane-bound lytic murein transglycosylase A |
| *PM1330* | PM1330 | Unknown | Inner membrane | SpII | NADH:ubiquinone oxidoreductase, putative |
| *apbE* | PM1334 | Cytoplasmic | Periplasm | SpII | thiamine biosynthesis lipoprotein |
| *PM1426* | PM1426 | Outer membrane | Outer membrane | SpI | phospholipase A |
| *PM1428* | PM1428 | Outer membrane | Outer membrane | SpI | iron-regulated Outer membrane protein, putative |
| *glpQ* | PM1444 | Outer membrane | Periplasm | SpII | glycerophosphodiester phosphodiesterase |
| *PM1448* | PM1448 | Cytoplasmic | cytoplasm | SpII | transcription accessory protein, putative, HI0568 |
| *xynC* | PM1451 | Unknown | Secreted | SpI | acetyl esterase |
| *cydD* | PM1474 | Inner membrane | Inner membrane | SpII | transport ATP binding protein |
| *vacJ* | PM1501 | Unknown | Outer membrane | SpII | vacJ lipoprotein homolog |
| *PM1509* | PM1509 | Unknown | Periplasm | SpII | unknown |
| *PM1514* | PM1514 | Unknown | No prediction | SpII | Outer membrane protein, putative |
| *plpE* | PM1517 | Unknown | No prediction | SpII | Outer membrane lipoprotein |
| *plpP* | PM1518 | Unknown | Inner membrane | SpII | Outer membrane lipoprotein |
| *PM1543* | PM1543 | Outer membrane | Periplasm | SpI | unknown |
| *hsf_2* | PM1570 | Unknown (This protein may have multiple localiation sites.) | Outer membrane | SpI | hsf homolog, locus encoding surface fibrils |
| *PM1578* | PM1578 | Unknown | Periplasm | SpII | immunogenic protein homolog |
| *PM1600* | PM1600 | Outer membrane | Outer membrane | SpI | organic solvent tolerance protein |
| *PM1603* | PM1603 | Unknown | Outer membrane | CYT | hypothetical, N. meningitidis |
| *PM1611* | PM1611 | Extracellular | Secreted | SpI | hypothetical E. coli |
| *PM1614* | PM1614 | Unknown | Outer membrane | SpII | Outer membrane antigenic lipoprotein B |
| *hasR* | PM1622 | Outer membrane | Outer membrane | CYT | heme receptor |
| *PM1717* | PM1717 | Outer membrane | Outer membrane | SpI | hypothetical component of transport and adhesion, AIDA-I |
| *PM1720* | PM1720 | Unknown | Secreted | SpII | lipoprotein, putative |
| *plpB* | PM1730 | Unknown | Outer membrane | SpII | Outer membrane lipoprotein 2 precursor |
| *PM1762* | PM1762 | Periplasmic | Periplasm | SpII | sugar ABC transporter, (sugar-binding protein), putative |
| *PM1805* | PM1805 | Unknown | Periplasm | SpII | unknown |
| *PM1808* | PM1808 | Outer membrane | Inner membrane | TMH | hypothetical HI0696 |
| *PM1809* | PM1809 | Outer membrane | Outer membrane | SpI | hypothetical HI0698 |
| *PM1819* | PM1819 | Outer membrane | No prediction | CYT | srfB, putative virulence factor |
| *PM1826* | PM1826 | Unknown | Secreted | SpII | unknown |
| *PM1827* | PM1827 | Unknown | Inner membrane | SpII | unknown |
| *PM1886* | PM1886 | Unknown | Outer membrane | SpII | small protein A precursor, putative, HI0838 |
| *PM1897* | PM1897 | Unknown | Periplasm | SpII | unknown |
| *PM1898* | PM1898 | Outer membrane | Outer membrane | CYT | peptidyl-prolyl cis-trans isomerase |
| *PM1905* | PM1905 | Unknown | Periplasm | SpII | carbonic anhydrase |
| *PM1979* | PM1979 | Outer membrane | Periplasm | CYT | peptidyl-prolyl cis-trans isomerse D, putative |
| *ibeB* | PM1980 | Outer membrane | Outer membrane | SpII | invasion gene locus |
| *PM1992* | PM1992 | Outer membrane | Outer membrane | SpI | Outer membrane antigen OMA87 |
| *skp* | PM1993 | Outer membrane | Outer membrane | SpI | skp, lipid A biosynthesis |
| *PM2006* | PM2006 | Unknown | Secreted | CYT | UDP-n-acetylglucosamine-peptide-n-acetylglucosaminyltransferase |
| *PM2008* | PM2008 | Unknown | Secreted | SpII | fimbrial biogenesis and twitching motility protein, putative |
| *PM2009* | PM2009 | Unknown | Secreted | CYT | membrane protein, putative, HI0367 |
